# Cannabis use and psychotic disorders in diverse settings in the Global South: findings from INTREPID II

**DOI:** 10.1017/S0033291723000399

**Published:** 2023-11

**Authors:** Joni Lee Pow, Casswina Donald, Marta di Forti, Tessa Roberts, Helen A. Weiss, Olatunde Ayinde, Sujit John, Bola Olley, Akin Ojagbemi, Georgina Miguel Esponda, Joseph Lam, Paramasivam Poornachandrika, Paola Dazzan, Fiona Gaughran, Palaniyandi Ponnusamy Kannan, Selvaraju Sudhakar, Jonathan Burns, Bonginkosi Chiliza, INTREPID Group, Alex Cohen, Oye Gureje, Rangaswamy Thara, Robin M. Murray, Craig Morgan, Gerard Hutchinson

**Affiliations:** 1 Department of Psychiatry, University of the West Indies, Saint Augustine, Trinidad; 2 Health Service and Population Research Department, Institute of Psychiatry, Psychology & Neuroscience, King's College London, London, UK; 3 Social, Genetic and Developmental Psychiatry, Institute of Psychiatry, Psychology & Neuroscience, King's College London, London, UK; 4 ESRC Centre for Society and Mental Health, King's College London, London, UK; 5 Centre for Global Mental Health, London School of Hygiene & Tropical Medicine, London, UK; 6 Department of Psychiatry, WHO Collaborating Centre for Research and Training in Mental Health, Neurosciences and Substance Abuse, University of Ibadan, Ibadan, Nigeria; 7 Department of Psychiatry, Schizophrenia Research Foundation, Chennai, India; 8 Institute of Mental Health, Madras Medical College, Kilpauk, Chennai, India; 9 National Institute for Health Research (NIHR) Mental Health Biomedical Research Centre at South London and Maudsley NHS Foundation Trust and King's College London, London, UK; 10 Psychological Medicine, Institute of Psychiatry, Psychology & Neuroscience, King's College London, London, UK; 11 Psychosis Studies Department, Institute of Psychiatry, Psychology & Neuroscience, King's College London, London, UK; 12 Department of Psychiatry, Rajiv Gandhi General Hospital and Madras Medical College, Chennai, India; 13 Department of Psychiatry, Chengelpet Medical College, Chengelpet, Tamil Nadu, India; 14 Mental Health Research Group, College of Medicine and Health, University of Exeter, Exeter, UK; 15 Department of Psychiatry, University of KwaZulu Natal, Durban, South Africa

**Keywords:** psychosis, schizophrenia, first-onset, cannabis, global mental health

## Abstract

**Background:**

Cannabis use has been linked to psychotic disorders but this association has been primarily observed in the Global North. This study investigates patterns of cannabis use and associations with psychoses in three Global South (regions within Latin America, Asia, Africa and Oceania) settings.

**Methods:**

Case–control study within the International Programme of Research on Psychotic Disorders (INTREPID) II conducted between May 2018 and September 2020. In each setting, we recruited over 200 individuals with an untreated psychosis and individually-matched controls (Kancheepuram India; Ibadan, Nigeria; northern Trinidad). Controls, with no past or current psychotic disorder, were individually-matched to cases by 5-year age group, sex and neighbourhood. Presence of psychotic disorder assessed using the Schedules for Clinical Assessment in Neuropsychiatry and cannabis exposure measured by the World Health Organisation Alcohol, Smoking and Substance Involvement Screening Test (ASSIST).

**Results:**

Cases reported higher lifetime and frequent cannabis use than controls in each setting. In Trinidad, cannabis use was associated with increased odds of psychotic disorder: lifetime cannabis use (adj. OR 1.58, 95% CI 0.99–2.53); frequent cannabis use (adj. OR 1.99, 95% CI 1.10–3.60); cannabis dependency (as measured by high ASSIST score) (adj. OR 4.70, 95% CI 1.77–12.47), early age of first use (adj. OR 1.83, 95% CI 1.03–3.27). Cannabis use in the other two settings was too rare to examine associations.

**Conclusions:**

In line with previous studies, we found associations between cannabis use and the occurrence and age of onset of psychoses in Trinidad. These findings have implications for strategies for prevention of psychosis.

The Global South (Africa, Asia, Latin America and the Caribbean) contains 85% of the world's population yet most of our knowledge on psychotic disorders is based on research in North America, Western Europe and Australasia (Hamilton & Sumnall, 2020; Hasan et al., 2020; Marconi, Di Forti, Lewis, Murray, & Vassos, 2016). INTREPID II is a programme of research incorporating incidence, case–control and cohort studies of psychoses in the Global South (Roberts et al., 2020). It has been designed to investigate variations in incidence, presentation, 2-year course and outcome, help-seeking and impact, as well as physical health with rates of untreated psychosis being used as a proxy for incidence (Morgan et al., 2023). The programme is based in three catchment areas of Kancheepuram in Tamil Nadu, India, Ibadan in Oyo State Nigeria and seven municipalities in northern Trinidad, with populations at risk of approximately 500 000 in each setting. These three settings encompass a range of economic levels, resources, ethnic groups and cultures.

A core aim of INTREPID II is to investigate the incidence and presentation of untreated psychotic disorders and associated risk factors in each setting. One of these putative risk factors is cannabis. The global trend towards decriminalisation and legalisation of cannabis use has brought increased attention to its association with psychosis (Murray & Hall, 2020). Previous research on cannabis and psychosis has typically explored the following: lifetime and frequency of use, age of first use, and potency of cannabis. Lifetime cannabis use has been associated with an increased risk for psychotic disorders, with more frequent use further increasing that risk (Di Forti et al., 2019; Henquet et al., 2005; van Os et al., 2002; Zammit, Allebeck, A, L, & L, 2002). Systematic reviews have observed that this occurs in a dose-response fashion with daily use associated with the greatest risk (Hasan et al., 2020; Marconi et al., 2016; Moore et al., 2007; Ortiz-Medina et al., 2018; van der Steur, Batalla, & Bossong, 2020). Much of this association is moderated by the potency of cannabis with higher tetrahydrocannabinol (THC) levels being linked to greater risks and worse outcomes (Di Forti et al., 2009; Di Forti et al., 2014; Di Forti et al., 2015; Di Forti et al., 2019; Quattrone et al., 2020). Using cannabis for the first time at a younger age has also been found to increase risk (Arseneault et al., 2002; Leadbeater, Ames, & Linden-Carmichael, 2019; Ortiz-Medina et al., 2018). Lastly, an earlier age of onset of psychosis amongst cannabis users has been extensively reported (Di Forti et al., 2014; Hasan et al., 2020; Helle et al., 2016; Large, Sharma, Compton, Slade, & Nielssen, 2011; Ongur, Lin, & Cohen, 2009; van der Steur et al., 2020). Intriguing findings from the EU-GEI study, reported high levels of cannabis use in the areas with high incidence of psychoses (Di Forti et al., 2019), raising the question of whether cannabis use contributes to the incidence and nature of psychotic disorders in diverse settings.

To date, most evidence on cannabis use and risk for psychosis comes from studies in the Global North and Australasia (Burkhard, Cicek, Barzilay, Radhakrishnan, & Guloksuz, 2021). Of the 10 studies in one meta-analysis (Marconi et al., 2016), none were from the Global South and of 36 studies in another review (Farris, Shakeel, & Addington, 2020) only one. This Brazilian study found cannabis use, especially earlier age of first use, to increase psychosis risk, but this study assessed prodromal symptoms and not psychosis (Serpa et al., 2018). Another study in Chile assessed the relative prevalence of schizophrenia in people treated for cannabis and cocaine use disorders, Although the study found the odds of having schizophrenia or other related disorders was almost five times greater among cannabis users than cocaine users it was unable to compare this to the odds of psychosis in the general population (Libuy, de Angel, Ibanez, Murray, & Mundt, 2018). Robust, population-based data from diverse settings are needed to better understand the contribution of cannabis use to psychotic disorders. In this paper, we present findings from a case–control study within INTREPID II that explored patterns of cannabis use between and within catchment areas in three diverse settings and examined the possible associations between cannabis exposure and risk of psychosis.

## Methods

### Design

Baseline recruitment and assessment of matched pairs of cases and controls was conducted between May 2018 and September 2020. In each setting, individuals with an untreated psychotic disorder were identified through a comprehensive case detection system. Case finding procedures were developed in the INTREPID I feasibility and pilot study and involved a multi-pronged approach including professional mental health services, folk providers and community key informants (Morgan et al., 2015, 2016). The predominantly rural catchment area in India was comprised of four sub-districts in Kancheepuram: Chengelpettu, Thiruporur, Uthiramerur and Maduranthakam. In Nigeria, the catchment area was comprised of three local government areas in Oyo State: Ibadan North East, Ibadan South East both urban and semi-rural Ona-Ara. In Trinidad, the catchment area was comprised of seven municipalities including both urban and rural areas: Port of Spain, Arima, Chaguanas, Tunapuna/Piarco, San Juan/Laventille, Diego Martin and Sangre Grande. These areas were chosen to capture economically and social diverse settings inclusive of both rural and urban areas. Description of the full INTREPID II protocol can be found in a previous publication (Roberts et al., 2020). A map of the catchment areas has been included in the online Supplement (Roberts et al., 2023).

### Inclusion and exclusion criteria

Inclusion criteria for cases were: between ages 18–64 years, resident in the catchment area, presence of ICD-10 psychotic disorder (including substance-induced psychosis), and not treated with antipsychotic medication for more than one continuous month prior to the start of case identification. Exclusion criteria were: transient psychotic symptoms resulting from acute intoxication, moderate or severe learning disability, and clinically manifest organic cerebral disorder, all as defined by ICD-10 criteria. Inclusion criteria for controls were: between ages 18–64 years, 5 years of age of index case, resident in catchment area, and same sex as index case. Exclusion criteria were: past or current ICD-10 psychotic disorder, moderate or severe learning disability, and clinically manifest organic cerebral disorder, as defined by ICD-10 criteria. As no sampling frame was available to randomly identify potential controls, the ten nearest neighbourhood households were mapped for each case, listing all residents by sex and age. All potential controls for the case (defined as the same sex and ± 5 years of age) were then approached in random order, until an eligible control was identified. When no match was identified the process was repeated. This approach was successfully piloted in all settings.

### Assessments

Screening for psychosis for both cases and controls was conducted using the Screening Schedule for Psychosis (Jablensky et al., 1992). For cases, the presence of psychosis was subsequently confirmed using the Schedules for Clinical Assessment in Neuropsychiatry (SCAN) (McGuffin, Farmer, & Harvey, 1991). Demographic data were collected using the MRC Sociodemographic Schedule. This included: employment (unemployed, inactive, student, employed), relationship (single, married, in a relationship, divorced, widowed), education (primary or less, secondary or higher) and ethnic group (Yoruba, Hausa, Igbo for Ibadan; Afro, Indo, Mixed/Other for Trinidad; Tamil, Telugu for Kancheepuram). Data on cannabis use was collected using the Alcohol, Smoking and Substance Involvement Screening Test (ASSIST) (Humeniuk et al., 2008) which screens for levels of substance use. The ASSIST consists of 8 questions covering various substances and allows for the generation of a risk score for each substance and is included in the online Supplement. The substances covered are tobacco products, alcohol, cannabis, cocaine, amphetamine type stimulants, inhalants, sedatives, hallucinogens and opioids. In each setting, assessments were conducted by trained local researchers and inter-rater reliability ratings were within acceptable margins of gold standard ratings developed by the Principal Investigators. Diagnoses were determined by consensus based on SCAN data and confirmed by a psychiatrist. Both cannabis and mental health history were self-reported and cross-referenced with hospital and/or clinic notes as well as a relative interview when available.

### Missing data

In line with all planned INTREPID II analyses, to handle missing data and avoid dropping observations, we used multiple imputation by chained equations (Azur, Stuart, Frangakis, & Leaf, 2011; Sterne et al., 2009; White, Royston, & Wood, 2011). The imputation model included all variables in the main analyses and several auxiliary variables. The following variables had missing data and were imputed: employment status, relationship status, education level, cannabis use ASSIST score, lifetime cannabis use, frequency of cannabis use, and tobacco use. Post-imputation analyses combined estimates across 25 imputed data sets using Rubin's rule (White et al., 2011).

### Analyses

This study sought to describe patterns of cannabis use among cases and controls in each setting, to estimate the effects of cannabis exposures on the odds of a psychotic disorder, and to explore whether cannabis use might account for any observed variation in incidence across settings. As the number of cannabis users in Ibadan and Kancheepuram were too small to allow reliable estimates, the analyses of effects of cannabis exposures on odds of psychosis were restricted to Trinidad. We used unconditional logistic regression, adjusted for matching variables, in preference to conditional, as such models can be more efficient and retain all participants in analyses where one of the matched case or control is missing data, thus limiting any loss of power (Pearce, 2016). In these unconditional logistic regression analyses the exposure variables were lifetime cannabis use (ASSIST question 1), high frequency of cannabis use defined as using cannabis weekly or more (ASSIST question 2), and the highest level of cannabis exposure indicated by an ASSIST risk score of 27 or more. A high score (27+) suggests dependence or a high risk of dependence and the individual is likely experiencing health, social, financial, legal and/or relationship problems due to their cannabis use. The fourth exposure variable is a lower age of first use defined as using cannabis for the first time at age 15 or below. As more reliable estimates were obtained when adjusting for matched variables, we adjusted for age, sex and ethnicity in one set of analyses and for additional potential confounders in another set of analyses (Pearce, 2016). Subsequently, odds ratios are reported for the four exposure variables, unadjusted, adjusted for age, sex and ethnicity in one column, and adjusted for age, sex, employment, education and relationship status in another column. Linear regression was used to assess the association between cannabis use and age of onset of psychosis amongst cases only, also adjusting for the previously listed confounders. STATA Version 16 was used.

## Results

### Incidence and case-control participants

We found wide variations in rates of untreated psychoses between settings in INTREPID II, with Trinidad having a higher rate [incidence rate (IR) = 59.1 per 100 000 person years, 95% CI 56.3–66.3] compared with the settings in Kancheepuram [IR = 20.7, 95% CI 18.3–23.3] and Ibadan (IR = 14.4, 95% CI 12.4–16.6). Clinical characteristics of cases varied across sites (Morgan et al., 2023) in line with variation observed in previous multi-site and multi-country studies (Jablensky et al., 1992; Quattrone et al., 2019; Quattrone et al., 2020).

We sought to identify all cases meeting inclusion criteria in each catchment area but were only able to assess a proportion for the case–control study. Cases were approached for recruitment in order of case identification. Cases who were assessed were, from the pool of all cases, those who consented to further full assessments. A comparison of the assessed and not assessed cases by core demographic and clinical variables is included in Table 1 of the online Supplement and shows no differences by age and gender (except for Kancheepuram), but some differences by diagnosis in Ibadan and Trinidad. The case–control population within INTREPID II comprised more than 200 age and sex matched pairs of cases and controls in each setting (225 Kancheepuram; 209 Ibadan; 212 Trinidad). A comparison of demographic characteristic for cases and controls can be found in Table 1. Cases had a median age of 44 years in Kancheepuram, 34 years in Ibadan and 31 years in Trinidad. In Kancheepuram 40% of the participants were men compared with 55% in Ibadan and 57% in Trinidad. These reflect differences in rates of psychoses by age and sex across settings. In all settings, cases were more likely than controls to be unemployed and single (not in a relationship). Compared with controls, cases had lower levels of education in Ibadan and Trinidad and similar levels of education in Kancheepuram. The study populations were ethnically homogenous in Kancheepuram (99% Tamil) and Ibadan (99% Yoruba), whereas in Trinidad was comprised primarily of three ethnic groups: 53% Afro-Trinidadian, 20% Indo-Trinidadian and 27% Mixed/Other-Trinidadian. The ethnic makeup of the control participants was similar to that of cases in Trinidad.

**Table 1.** Demographic characteristics of cases and matched controls in each INTREPID setting

Characteristic	Study participants, no. (%)					
	Ibadan		Trinidad		Kancheepuram	
	Cases (*n* = 209)	Controls (*n* = 209)	Cases (*n* = 212)	Controls (*n* = 212)	Cases (*n* = 225)	Controls (*n* = 225)
Median age, years (IQR)	34 (26–41)	33 (25–41)	31 (24–39)	31 (23–38)	44 (34–50)	42 (35–51)
Gender						
Male	116 (55)	116 (55)	122 (57)	122 (57)	90 (40)	90 (40)
Female	93 (44)	93 (44)	90 (42)	90 (42)	135 (60)	135 (60)
Employment						
Unemployed	127 (61)	11 (5)	117 (56)	42 (20)	174 (77)	86 (38)
Inactive	2 (1)	1 (0.5)	16 (8)	1 (0.5)	0 (-)	2 (0.9)
Student	12 (6)	12 (6)	7 (3)	8 (4)	5 (2)	3 (1)
Employed	66 (31)	183 (87)	69 (32)	161 (76)	46 (20)	134 (59)
Relationship						
Single	96 (46)	34 (16)	124 (59)	88 (41)	23 (10)	62 (28)
Married	43 (21)	101 (48)	41 (20)	65 (31)	171 (76)	105 (47)
In relationship	23 (11)	58 (28)	30 (14)	47 (22)	0 (-)	1 (0.4)
Divorced	39 (19)	10 (5)	11 (5)	8 (4)	6 (3)	26 (12)
Widowed	8 (4)	6 (3)	3 (1)	4 (2)	25 (11)	31 (14)
Education						
Primary or less	53 (25)	42 (20)	66 (32)	31 (15)	138 (61)	138 (61)
Secondary or higher	156 (75)	167 (80)	141 (66)	181 (85)	87 (39)	87 (39)
Ethnic group						
Yoruba	204 (99)	205 (99)	–	–	–	–
Hausa	2 (0.9)	0 (–)	–	–	–	–
Igbo	1 (0.5)	3 (1)	–	–	–	–
Afro- Trinidadian	–	–	112 (53)	111 (52)	–	–
Indo- Trinidadian	–	–	41 (19)	45 (21)	–	–
Mixed/Other-Trinidadian	–	–	59 (28)	56 (25)	–	–
Tamil	–	–	–	–	225 (100)	224 (99)
Telegu	–	–	–	–	0 (–)	1 (0.4)

There was wide variation between the settings in cannabis exposure with most of the participants in Kancheepuram never using cannabis, less than a quarter (21%) of participants having used cannabis in Ibadan and over two-thirds in Trinidad (68%). In all settings, cases were more likely than controls to report lifetime cannabis use (72% *v.* 66% in Trinidad; 30% *v.* 13% in Ibadan; 4% *v.* 0.5% in Kancheepuram) and frequent use (27% *v.* 21% in Trinidad; 13% *v.* 4% in Ibadan; 0.9% *v.* 0.4%) (Table 2).

**Table 2.** Prevalence of cannabis exposure by case control status in each INTREPID setting

Exposure variable	Study participants, No. (%)					
	Trinidad		Ibadan		Kancheepuram	
	Cases	Controls	Cases	Controls	Cases	Controls
Lifetime cannabis use	145/201 (72.1)	136/209 (66.1)	60/203 (30.6)	28/209 (13.4)	9/225 (4.0)	1/225 (0.5)
Frequent cannabis use (⩾once per week)	55/201 (27.4)	43/209 (20.6)	27/203 (13.3)	9/209 (4.3)	2/225 (0.9)	1/225 (0.4)
High ASSIST risk score 27+	21/201 (10.4)	7/209 (3.3)	10/203 (4.9)	2/209 (0.9)	1/225 (0.4)	1/225 (0.4)
Age first use ⩽15	62/200 (31.0)	50/207 (24.2)	19/202 (9.4)	8/209 (3.8)	0/225	0/225

Using controls as a proxy for population estimates in the same demographic groups as cases, frequent cannabis users in Ibadan and Trinidad were more likely to be younger, men and single compared with never users. In Trinidad, cannabis users were more likely to be of Afro-Trinidadian and Mixed/other-Trinidadian ethnicity compared with never users. Details on the demographic characteristics of frequent cannabis users compared with never users in these settings can be found in Table 2 in the online Supplement. Due to the low prevalence of cannabis use in Kancheepuram, a comparison of frequent users with never users was not meaningful.

All four cannabis exposure variables were associated with increased odds of psychotic disorder in Trinidad (Table 3). The prevalence of cannabis use was too low in the other settings to enable reliable estimates. Multiple imputations were used to handle missing data in Trinidad, where 5% (11/212) of cases and 2% (3/212) of controls had missing data for all cannabis exposure variables. A table comparing the observed characteristics between participants with complete and incomplete data can be found in Table 3 in the online Supplement.

**Table 3.** Risk of psychotic disorder associated with cannabis exposure in Trinidad

Exposure variable	OR (95% CI)		
	Unadjusted	Adjusted^a^	Adjusted^b^
Lifetime cannabis use	1.37 (0.90–2.09)	1.58 (0.99–2.53)	1.67 (0.96–2.91)
Frequent cannabis use (⩾once per week)	1.70 (1.00–2.89)	1.99 (1.10–3.60)	2.25 (1.13–4.47)
High ASSIST risk score 27+	3.87 (1.53–9.75)	4.70 (1.77–12.47)	4.25 (1.44–12.52)
Age first use ⩽15	1.56 (0.94–2.61)	1.83 (1.03–3.27)	1.87 (0.95–3.67)

a Adjusted for age, sex and ethnicity.

b Adjusted for age, sex, ethnicity, employment, education and relationship.

In Trinidad, compared with never users, lifetime cannabis use was associated with moderate increased odds of psychosis (adjusted OR 1.58, 95% CI 0.99–2.53) and frequent use increased this (adjusted OR 1.99, 95% CI 1.10–3.60). The possibility that the onset of psychosis preceded cannabis use was explored. However, it was found that only 9% (5/55) of Trinidadian cases with frequent cannabis use reported an onset of psychosis that preceded the age of first cannabis use. The highest level of cannabis exposure (indicated by an ASSIST score ⩾27) was associated with the strongest association with psychosis (adjusted OR 4.70, 95% CI 1.77–12.47).

In Trinidad, using cannabis for the first time at age 15 or younger was associated with an adjusted OR of 1.83 (95% CI 1.03–3.27) compared with never users. In addition, early first use appeared to be linked to frequent adult use. Approximately half of the Trinidadian participants (52% (26/50) controls, 48% (30/62) cases) whose first cannabis use was at age 15 or younger went on to become frequent adult users.

There was variation in the median age of onset (Trinidad 24.74 years, Ibadan 28.50, Kancheepuram 33.37). In Trinidad, cannabis use was associated with a 4 year earlier onset of psychosis [mean age 25 *v.* 30 years, unadjusted: *β* = −4.69, *t*(212) = −2.51, adjusted^a^: *β* = −3.48, *t*(212) = −1.72, adjusted^b^: *β* = −1.97, *t*(212) = −0.97]. Within Trinidad, higher levels of cannabis exposure also appeared to be linked to lower median age of onset in years (never users: 28.49 years, lifetime users: 23.77, frequent users: 22.99, high ASSIST score: 22.41).

## Discussion

**Fig. 1.**
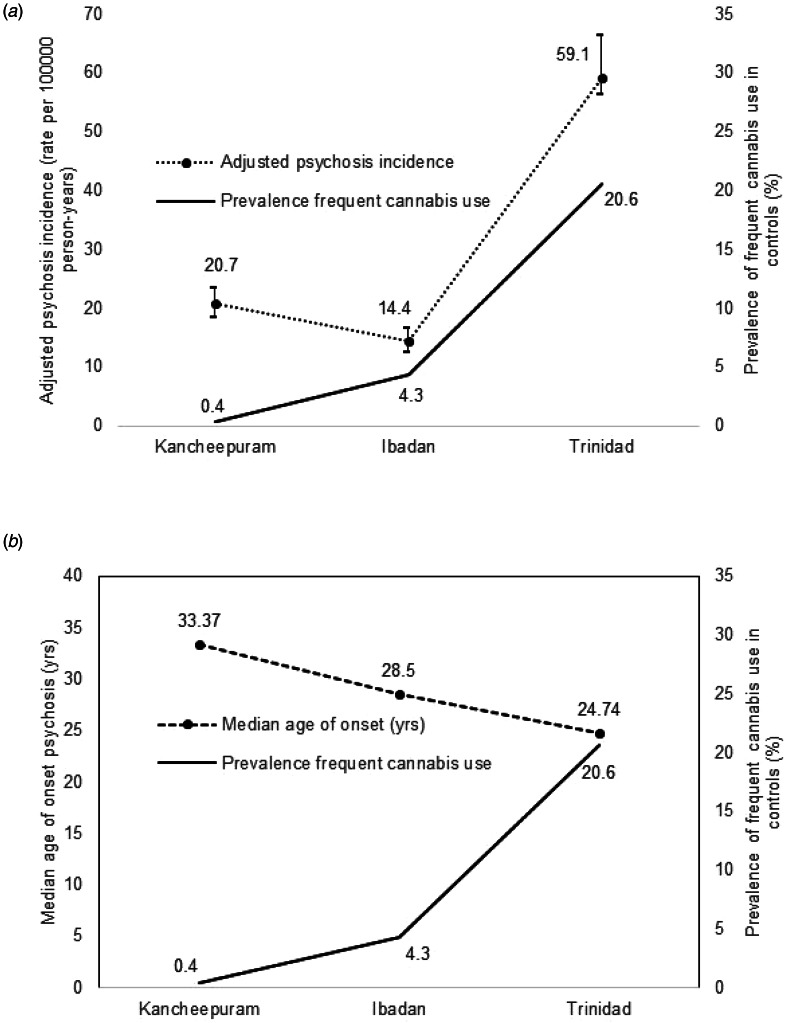
(*a*) Adjusted incidence rates for psychosis in the INTREPID II sites plotted against the prevalence of frequent cannabis use in matched controls and median age of onset of psychosis. (*b*) Median age of onset of psychosis plotted against the prevalence of frequent cannabis use in matched controls.

This study is the first of its kind to examine any association between cannabis use and psychosis in the Global South. We observed wide variation in cannabis use between the three settings with the highest levels in Trinidad. Despite this variation, cannabis exposure within each setting was more prevalent amongst cases compared with controls. Our findings in Trinidad confirm previous findings from the Global North that increased levels of cannabis exposure is associated with increased odds of a psychotic disorder, i.e., lifetime use associated with a 1.5 times increased odds, both frequent use and early adolescent use with around a two-fold increased odds, and a high ASSIST risk score with an almost five-fold increased odds. Within Trinidad, lifetime cannabis use was also linked to an earlier onset of psychosis. Variation between settings adds support to this finding as settings with more lifetime cannabis use had a lower age onset of psychosis. Additionally, within Trinidad, as cannabis exposure increased the median age of onset correspondingly lowered (never use: 28.49 years, lifetime use: 23.77, frequent use: 22.99, high ASSIST risk score: 22.41). Overall, our findings indicate that incidence rates are higher and age of onset lower in settings with more prevalent cannabis use (Fig. 1a and 1b).

There are some limitations to our study. High co-use with tobacco in our Trinidadian study population made it difficult to estimate the effects of cannabis and tobacco independently. Furthermore, it is unlikely that the observed higher incidence rates in Trinidad can be solely attributed to frequent cannabis use. Cannabis potency was not measured in our study, but it has previously been observed as an important contributory factor (Di Forti et al., 2009; Di Forti et al., 2014; Di Forti et al., 2019). So called ‘high potency’ cannabis in recent UK-based studies is characterised by 12–18% THC content. Common strains of Trinidadian cannabis are estimated to range from 19–26% THC (Espinet, 2019). In Trinidad, where higher rates of psychoses and cannabis prevalence were observed, there may be common risk factors driving both. Similarly, in the other sites, there may be protective factors that contribute to lower rates of both psychoses and cannabis prevalence such as religiosity and family support. We cannot exclude the possibility that other putative factors may be contributing to the high rates observed in Trinidad e.g. high levels of trauma or urbanicity. These other factors and their interaction with cannabis use will be the focus of future analyses. Because we have not done so yet and because much of what is known about psychoses suggests multiple factors being involved in onset, our claims on the impact of cannabis remain tentative. Nevertheless, of these known risk factors, cannabis remains the most modifiable and hence important. Although significant measures were taken to pilot and develop thorough case-finding methods, between-setting variation in incidence rates may reflect differences in case finding as Trinidad has well-developed accessible mental health services compared with the other settings. In Ibadan and Kancheepuram, case finding was as comprehensive as possible, i.e. where trained researchers conducted regular checks with community informants to identify cases and ensured that informants understood what was meant by psychosis but it remains possible that some cases were nonetheless missed. There may have also been differences in the willingness to report cannabis use especially in Kancheepuram where family was often present during data collection. Although the prevalence of cannabis use was lower in Kancheepuram and Ibadan, these findings represent the catchment areas studied and should not be taken as representative of cannabis use in the wider country from where the participants were selected. In particular, the catchment area in India is predominantly rural and cannabis use has been found to differ considerably for an urban population. For instance, a 2003 study in Bangalore, India observed that high cannabis use was associated with psychiatric disorders especially psychosis and mania (Sarkar, Murthy, & Singh, 2003). Further, findings from Mendelian randomisation studies suggest that there may be a bi-directional association between cannabis use and psychosis with a shared genetic risk contributing to both psychosis risk and tendency to use cannabis (Gage et al., 2017; Pasman et al., 2018; Vaucher et al., 2018). A more recent review of various genetic methods concludes that relatively reliable evidence indicate that genetic risk contributes to the cannabis-schizophrenia association but good evidence also indicates that genetic risk does not explain all of this association (Gillespie & Kendler, 2021). This said, of the 145 cases in Trinidad who used cannabis in their lifetime, most (84%) first used cannabis before the age of psychosis onset. However, it is possible that prodromal symptoms increased the likelihood of cannabis use, e.g. to self-medicate, pre-onset and this adds a note of caution in inferring a causal effect from our findings. Finally, while the amount of missing data on cannabis use was low (in Trinidad, 5% of cases (e.i., 11 of 212) and 1% of controls (i.e. 3 of 212)), we nonetheless imputed this data, rather than removing participants, to ensure a more consistent approach with further planned analyses.

The results from this study suggest that cannabis use is high in Trinidad compared with international estimates. In the EU-GEI study, the proportion of controls that were daily cannabis users in London and Amsterdam was 11.7% and 13.1% respectively (Di Forti et al., 2019). In Trinidad, 19.6% of controls were daily cannabis users, albeit some of this difference may be due to controls in INTREPID being age and sex matched with cases. The high prevalence of cannabis use and its associations with psychosis highlights the need for mental health services in Trinidad to identify cannabis use amongst patients and provide appropriate addiction services. Furthermore, our data indicate that the desire to stop cannabis use is present. Ten per cent of controls (21/209) and 27% of cases (54/201) responded yes to the ASSIST item ‘Have you ever tried and failed to control, cut down or stop using?’, indicating a greater attempt amongst cases compared with controls for cannabis cessation. However, high co-use with tobacco is present as 17% (35/201) of cases compared with 12% (26/209) of controls use both cannabis and tobacco daily, so cannabis cessation strategies would need to also address nicotine addiction. Early age of use is also a concern as almost a quarter of Trinidadian controls first used cannabis before or at age 15 (compared with 13.7% of the EU-GEI controls) and approximately half of all Trinidadian early users went on to become frequent adult users. The possession of cannabis is illegal in Ibadan and Kancheepuram but cannabis was decriminalised in Trinidad in December 2019 (Dangerous Drugs Amendment Act, 2019) allowing a person to have on their possession up to 30 grams of cannabis. This may or may not have contributed to the high prevalence of cannabis use but it potentially makes it more challenging to target reduction in adult frequent use. Based on the prevalence of early first use, and the known risks of substance use in younger populations, targeting the prevention of adolescent cannabis use is an important approach. We recommend the development and implementation of a public education programme on the risks associated with cannabis use.

## Conclusion

We observed wide variations in the incidence of psychosis and prevalence of cannabis use between settings, with the highest rates for both in Trinidad. Here we found an association between cannabis use and risk of psychotic disorder. Cannabis use may therefore account, in part, for differential rates of psychoses and between-setting differences in age of onset of psychosis. Similar to international findings, incidence rates in our study are higher and age of onset lower in the settings with more prevalent cannabis use. The high prevalence of cannabis use in Trinidad calls for further investigation into cannabis use and its associated risks within the Caribbean.

## Supporting information

Lee Pow et al. supplementary material Lee Pow et al. supplementary material

## Supplementary material

The supplementary material for this article can be found at https://doi.org/10.1017/S0033291723000399.
