# Supplementary material for: Cannabis use and psychotic disorders in diverse settings in the Global South: findings from INTREPID II
Source: Psychol Med. 2023 Mar 23;53(15):7062–9. doi: 10.1017/S0033291723000399 (PMC10719629; doi:10.1017/S0033291723000399)
Supplement: Lee Pow et al. supplementary material [file S0033291723000399sup001.docx]

**Supplementary Material**

Table 1. Comparison of not assessed and assessed cases by core demographic and clinical variables.

|  | |  | Kancheepuram  n 268 | | | Ibadan  n 276 | | | Trinidad  n 574 | | |
| --- | --- | --- | --- | --- | --- | --- | --- | --- | --- | --- | --- |
|  | |  | Not Assessed | Assessed | p | Not Assessed | Assessed | p | Not Assessed | Assessed | p |
| Age at detection | | mean (sd) | 38.8 (11.9) | 42.4 (11.4) | 0.062 | 33.9 (11.4) | 34.7 (10.3) | 0.606 | 32.5 (11.7) | 32.7 (11.0) | 0.836 |
| Sex | |  |  |  |  |  |  |  |  |  |  |
|  | Men | n (%) | 24 (55.8) | 90 (40.0) | 0.055 | 37 (55.2) | 116 (55.5) | 0.968 | 229 (59.6) | 122 (57.6) | 0.620 |
|  | Women | n (%) | 19 (44.2) | 135 (60.0) |  | 30 (44.8) | 93 (44.5) |  | 155 (40.4) | 90 (42.5) |  |
| Diagnosis | |  |  |  |  |  |  |  |  |  |  |
|  | Non affective | n (%) | 43 (100.0) | 216 (96.0) | 0.182 | 42 (62.7) | 192 (91.9) | < 0.001 | 317 (82.6) | 131 (61.8) | < 0.001 |
|  | Affective | n (%) | 0 (0.0) | 9 (4.0) |  | 25 (27.3) | 17 (8.1) |  | 67 (17.5) | 81 (38.2) |  |

Table 2. Demographic characteristics of frequent cannabis users and never users in controls within Trinidad and Ibadan.

|  | **Control participants, No. (%)** | | | |
| --- | --- | --- | --- | --- |
|  | **Ibadan** | | **Trinidad** | |
| **Characteristic** | **Frequent cannabis users (n=9)** | **Never users (n=181)** | **Frequent cannabis users (n=43)** | **Never users (n=73)** |
| Median age, years (IQR) | 28  (25-32) | 34  (26-42) | 26  (22-34) | 33  (25-43) |
| Gender |  |  |  |  |
| Male | 9 (100) | 88 (49) | 34 (79) | 28 (38) |
| Female | 0 (-) | 93 (51) | 9 (21) | 45 (62) |
| Employment |  |  |  |  |
| Unemployed | 0 (-) | 11 (6) | 11 (26) | 14 (19) |
| Inactive | 0 (-) | 1 (0.6) | 0 (-) | 1 (1) |
| Student | 0 (-) | 11 (6) | 0 (-) | 4 (5) |
| Employed | 9 (100) | 157 (87) | 32 (74) | 54 (74) |
| Relationship |  |  |  |  |
| Single | 4 (44) | 24 (13) | 18 (42) | 23 (31) |
| Married | 1 (11) | 92 (51) | 9 (21) | 29 (40) |
| In relationship | 3 (33) | 51 (28) | 14 (33) | 15 (21) |
| Divorced | 1 (11) | 8 (4) | 2 (5) | 3 (4) |
| Widowed | 0 (-) | 6 (3) | 0 (-) | 3 (4) |
| Education |  |  |  |  |
| Primary or less | 1 (11) | 37 (20) | 8 (19) | 10 (14) |
| Secondary or higher | 8 (89) | 144 (80) | 35 (81) | 63 (86) |
| Ethnic Group |  |  |  |  |
| Yoruba | 9 (100) | 177 (98) | - | - |
| Hausa | 0 (-) | 0 (-) | - | - |
| Igbo | 0 (-) | 1 (2) | - | - |
| Afro- Trinidadian | - | - | 26 (60) | 33 (45) |
| Indo- Trinidadian | - | - | 4 (9) | 24 (33) |
| Mixed/Other-Trinidadian | - | - | 13 (30) | 16 (22) |

Table 3. Demographics characteristics of cases with missing cannabis data compared with cases with complete data in Trinidad.

|  | **Study participants Trinidad, No. (%)** | | | |
| --- | --- | --- | --- | --- |
|  | **Missing cannabis data** | | **Complete data** | |
| **Characteristic** | **Cases**  **(n=11)** | **Controls (n=3)** | **Cases**  **(n=201)** | **Controls (n=209)** |
| Median age, years (IQR) | 36  (26-43) | 29  (22-35) | 30  (24-39) | 31  (23-38) |
| Gender |  |  |  |  |
| Male | 5 (45) | 2 (67) | 117 (58) | 120 (57) |
| Female | 6 (55) | 1 (33) | 84 (42) | 89 (43) |
| Employment |  |  |  |  |
| Unemployed | 6 (55) | 1 (33) | 111 (55) | 41 (20) |
| Inactive | 0 (-) | 0 (-) | 16 (8) | 1 (0.5) |
| Student | 0 (-) | 0 (-) | 7 (3) | 8 (4) |
| Employed | 2 (18) | 2 (67) | 67 (33) | 159 (76) |
| Missing | 3 (27) | 0 (-) | 0 (-) | 0 (-) |
| Relationship |  |  |  |  |
| Single | 6 (55) | 1 (33) | 118 (59) | 87 (42) |
| Married | 1 (0.1) | 2 (67) | 41 (20) | 63 (30) |
| In relationship | 0 (-) | 0 (-) | 29 (14) | 47 (22) |
| Divorced | 1 (0.1) | 0 (-) | 10 (5) | 8 (4) |
| Widowed | 0 (-) | 0 (-) | 3 (1) | 4 (2) |
| Missing | 3 (27) | 0 (-) | 0 (-) | 0 (-) |
| Education |  |  |  |  |
| Primary or less | 4 (36) | 0 (-) | 62 (31) | 31 (15) |
| Secondary or higher | 2 (18) | 3 (100) | 139 (69) | 178 (85) |
| Missing | 5 (45) | 0 (-) | 0 (-) | 0 (-) |
| Ethnic group |  |  |  |  |
| Afro- Trinidadian | 4 (36) | 2 (67) | 108 (54) | 109 (52) |
| Indo- Trinidadian | 4 (36) | 0 (-) | 37 (18) | 45 (21) |
| Mixed/Other-Trinidadian | 3 (27 | 1 (33) | 56 (28) | 55 (26) |

Fig. 1 Maps of INTREPID II catchment areas in India, Nigeria and Trinidad

Black borders indicate the boundaries of the total catchment area in each setting and the administrative areas within these.


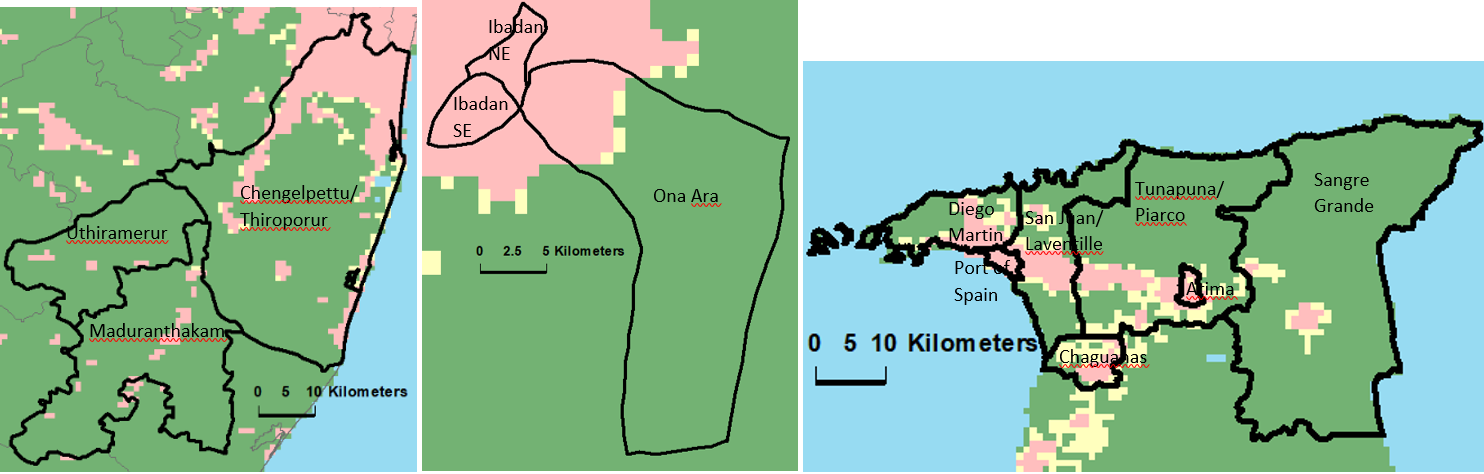


*Note*. Pink indicates urban areas (Class 30: “Urban Centre”, Class 23: “Dense Urban Cluster”, and Class 22: “Semi-dense Urban Cluster”), yellow indicates peri-urban areas (Class 21: “Suburban or peri-urban”), while green indicates rural areas (Class 13: “Rural cluster”, Class 12: “Low Density Rural”, and Class 11: “Very low density rural”).

Alcohol, Smoking and Substance Involvement Screening Test (ASSIST)

INTRODUCTION *(Please read to patient)*

Thank you for agreeing to take part in this brief interview about alcohol, tobacco products and other

drugs. I am going to ask you some questions about your experience of using these substances across

your lifetime and in the past three months. These substances can be smoked, swallowed, snorted,

inhaled, injected or taken in the form of pills (show drug card).

Some of the substances listed may be prescribed by a doctor (like amphetamines, sedatives, pain

medications). For this interview, we will not record medications that are used as prescribed by your

doctor. However, if you have taken such medications for reasons other than prescription, or taken them more frequently or at higher doses than prescribed, please let me know. While we are also interested in knowing about your use of various illicit drugs, please be assured that information on such use will be treated as strictly confidential.

1. In your life, which of the following substances have you ever used? *(NON-MEDICAL USE ONLY)*

Yes = 3

No = 0

a. Tobacco products (cigarettes, chewing tobacco, cigars, etc.)

b. Alcoholic beverages (beer, wine, spirits, etc.)

c. Cannabis (marijuana, pot, grass, hash, etc.)

d. Cocaine (coke, crack, etc.)

e. Amphetamine type stimulants (speed, diet pills, ecstasy, etc.)

f. Inhalants (nitrous, glue, petrol, paint thinner, etc.)

g. Sedatives or Sleeping Pills (Valium, Serepax, Rohypnol, etc.)

h. Hallucinogens (LSD, acid, mushrooms, PCP, Special K, etc.)

i. Opioids (heroin, morphine, methadone, codeine, etc.)

j. Other - specify: ________________

Probe if all answers are negative: “Not even when you were in school?”

*If "No" to all items, stop interview. If "Yes" to any of these items, ask Question 2 for each substance ever used.*

1. In the past three months, how often have you used the substances you mentioned *(FIRST DRUG, SECOND DRUG, ETC)*?

Never = 0

Once or twice = 2

Monthly = 3

Weekly = 4

Daily or almost daily = 6

a. Tobacco products (cigarettes, chewing tobacco, cigars, etc.)

b. Alcoholic beverages (beer, wine, spirits, etc.)

c. Cannabis (marijuana, pot, grass, hash, etc.)

d. Cocaine (coke, crack, etc.)

e. Amphetamine type stimulants (speed, diet pills, ecstasy, etc.)

f. Inhalants (nitrous, glue, petrol, paint thinner, etc.)

g. Sedatives or Sleeping Pills (Valium, Serepax, Rohypnol, etc.)

h. Hallucinogens (LSD, acid, mushrooms, PCP, Special K, etc.)

i. Opioids (heroin, morphine, methadone, codeine, etc.)

j. Other - specify: ________________

*If used (a), please specify the type of tobacco used:*

*__________________________________________________*

*If used (a), please specify the average number of cigarettes (or other tobacco products) used per day:*

*__________________________________________________*

*If used (b), please specify the type of alcohol usually drunk:*

*__________________________________________________*

*If used (b), please specify the number of alcoholic drinks consumed on a typical day when drinking (see STEPS flashcards for definition of a “standard drink”):*

*__________________________________________________*

*If used (c), please specify the type of cannabis used:*

*__________________________________________________*

*If "Never" to all items in Question 2, skip to Question 6.*

*If any substances in Question 2 were used in the previous three months, continue with Questions 3, 4 & 5 for each substance used.*

1. During the past three months, how often have you had a strong desire or urge to use *(FIRST DRUG, SECOND DRUG, ETC)*?

Never = 0

Once or twice = 3

Monthly = 4

Weekly = 5

Daily or almost daily = 6

a. Tobacco products (cigarettes, chewing tobacco, cigars, etc.)

b. Alcoholic beverages (beer, wine, spirits, etc.)

c. Cannabis (marijuana, pot, grass, hash, etc.)

d. Cocaine (coke, crack, etc.)

e. Amphetamine type stimulants (speed, diet pills, ecstasy, etc.)

f. Inhalants (nitrous, glue, petrol, paint thinner, etc.)

g. Sedatives or Sleeping Pills (Valium, Serepax, Rohypnol, etc.)

h. Hallucinogens (LSD, acid, mushrooms, PCP, Special K, etc.)

i. Opioids (heroin, morphine, methadone, codeine, etc.)

j. Other - specify: ________________

1. During the past three months, how often has your use of *(FIRST DRUG, SECOND DRUG, ETC*) led to health, social, legal or financial problems?

Never = 0

Once or twice = 4

Monthly = 5

Weekly = 6

Daily or almost daily = 7

a. Tobacco products (cigarettes, chewing tobacco, cigars, etc.)

b. Alcoholic beverages (beer, wine, spirits, etc.)

c. Cannabis (marijuana, pot, grass, hash, etc.)

d. Cocaine (coke, crack, etc.)

e. Amphetamine type stimulants (speed, diet pills, ecstasy, etc.)

f. Inhalants (nitrous, glue, petrol, paint thinner, etc.)

g. Sedatives or Sleeping Pills (Valium, Serepax, Rohypnol, etc.)

h. Hallucinogens (LSD, acid, mushrooms, PCP, Special K, etc.)

i. Opioids (heroin, morphine, methadone, codeine, etc.)

j. Other - specify: ________________

1. During the past three months, how often have you failed to do what was normally expected of you because of your use of *(FIRST DRUG, SECOND DRUG, ETC*)?

Never = 0

Once or twice = 5

Monthly = 6

Weekly = 7

Daily or almost daily = 8

a. Tobacco products (cigarettes, chewing tobacco, cigars, etc.)

b. Alcoholic beverages (beer, wine, spirits, etc.)

c. Cannabis (marijuana, pot, grass, hash, etc.)

d. Cocaine (coke, crack, etc.)

e. Amphetamine type stimulants (speed, diet pills, ecstasy, etc.)

f. Inhalants (nitrous, glue, petrol, paint thinner, etc.)

g. Sedatives or Sleeping Pills (Valium, Serepax, Rohypnol, etc.)

h. Hallucinogens (LSD, acid, mushrooms, PCP, Special K, etc.)

i. Opioids (heroin, morphine, methadone, codeine, etc.)

j. Other - specify: ________________

*Ask Questions 6, 7 & 8 for all substances ever used (i.e. those endorsed in Question 1)*

1. Has a friend or relative or anyone else ever expressed concern about your use of *(FIRST DRUG, SECOND DRUG, ETC.)?*

No, Never = 0

Yes, in the past 3 months = 6

Yes, but not in the past 3 months = 3

a. Tobacco products (cigarettes, chewing tobacco, cigars, etc.)

b. Alcoholic beverages (beer, wine, spirits, etc.)

c. Cannabis (marijuana, pot, grass, hash, etc.)

d. Cocaine (coke, crack, etc.)

e. Amphetamine type stimulants (speed, diet pills, ecstasy, etc.)

f. Inhalants (nitrous, glue, petrol, paint thinner, etc.)

g. Sedatives or Sleeping Pills (Valium, Serepax, Rohypnol, etc.)

h. Hallucinogens (LSD, acid, mushrooms, PCP, Special K, etc.)

i. Opioids (heroin, morphine, methadone, codeine, etc.)

j. Other - specify: ________________

1. Have you ever tried and failed to control, cut down or stop using *(FIRST DRUG, SECOND DRUG, ETC.)?*

No, Never = 0

Yes, in the past 3 months = 6

Yes, but not in the past 3 months = 3

a. Tobacco products (cigarettes, chewing tobacco, cigars, etc.)

b. Alcoholic beverages (beer, wine, spirits, etc.)

c. Cannabis (marijuana, pot, grass, hash, etc.)

d. Cocaine (coke, crack, etc.)

e. Amphetamine type stimulants (speed, diet pills, ecstasy, etc.)

f. Inhalants (nitrous, glue, petrol, paint thinner, etc.)

g. Sedatives or Sleeping Pills (Valium, Serepax, Rohypnol, etc.)

h. Hallucinogens (LSD, acid, mushrooms, PCP, Special K, etc.)

i. Opioids (heroin, morphine, methadone, codeine, etc.)

j. Other - specify: ________________

1. How old were you when you first used *(FIRST DRUG, SECOND DRUG, ETC.)?*

a. Tobacco products (cigarettes, chewing tobacco, cigars, etc.)

b. Alcoholic beverages (beer, wine, spirits, etc.)

c. Cannabis (marijuana, pot, grass, hash, etc.)

d. Cocaine (coke, crack, etc.)

e. Amphetamine type stimulants (speed, diet pills, ecstasy, etc.)

f. Inhalants (nitrous, glue, petrol, paint thinner, etc.)

g. Sedatives or Sleeping Pills (Valium, Serepax, Rohypnol, etc.)

h. Hallucinogens (LSD, acid, mushrooms, PCP, Special K, etc.)

i. Opioids (heroin, morphine, methadone, codeine, etc.)

1. Have you ever used any drug by injection? *(NON-MEDICAL USE ONLY)*

No, Never = 0

Yes, in the past 3 months = 6

Yes, but not in the past 3 months = 3
